# Acute effects of immediate-release methylphenidate on QbTest performance in adults with untreated ADHD and longstanding polysubstance use: a randomized double-blind placebo-controlled crossover study

**DOI:** 10.1186/s12888-026-08717-3

**Published:** 2026-11-11

**Authors:** Monica Löhman, Blanca Domingo, Lennart Jansson

**Affiliations:** 1 Psychiatric Clinic, Region Västmanland, Västerås, 721 89 Sweden; 2 Centre for Clinical Research, Uppsala University, Västerås, Sweden

**Keywords:** Polysubstance use, ADHD, Methylphenidate, Randomized controlled trial (RCT)

## Abstract

**Background:**

Although methylphenidate (MPH) is a first-line treatment for adult attention-deficit/hyperactivity disorder (ADHD) in Europe, evidence in individuals with co-occurring ADHD and substance use disorder (SUD) remains limited, particularly in those with longstanding polysubstance use. This study examined the acute effects of immediate-release methylphenidate (IR-MPH) versus placebo on cognitive performance in this population.

**Methods:**

In this randomized, double-blind, placebo-controlled crossover study, 39 adults with untreated ADHD and longstanding polysubstance use completed the QbTest before and 80 min after a single 20-mg dose of IR-MPH and placebo on separate study days. Objective outcomes were the cardinal QbTest parameters QbActivity, QbInattention, and QbImpulsivity. Subjective outcomes included self-rated expected performance, perceived performance, perceived mental effort, perceived benefit from the study medication, and task comprehension. Repeated-measures analyses of variance (ANOVA) and paired-samples t-tests were used to compare outcomes across conditions.

**Results:**

Compared with baseline, IR-MPH significantly improved QbInattention [mean difference 1.00, 95% CI (0.55–1.45), *p* < 0.001], QbActivity [mean difference 0.83, 95% CI (0.37–1.28), *p* < 0.001], and QbImpulsivity [mean difference 0.83, 95% CI (0.12–1.55), *p* < 0.001]. These effects remained significant after adjustment for placebo effects. Placebo was associated with significant improvement only in reaction time [mean difference 35.15, 95% CI (2.37–67.84), *p* < 0.029]. Expected performance and perceived performance improved after IR-MPH but not after placebo, whereas mental effort decreased after both conditions. Participants reported greater perceived benefit from IR-MPH than from placebo. No QbTest order effects were observed.

**Conclusions:**

IR-MPH was associated with short-term improvements in objective QbTest performance in adults with untreated ADHD and longstanding polysubstance use, whereas placebo effects were limited on objective outcomes and were more evident in subjective experience. These findings support the inclusion of objective assessments alongside self-report when evaluating early treatment response in clinically complex adults with ADHD and co-occurring SUD.

**Trial registration:**

NCT02477280 in ClinicalTrial.gov, registered on June 22, 2015.

## Introduction

Substance use disorder (SUD) is characterized by a maladaptive pattern of cognitive, behavioral, and physiological symptoms that indicate continued substance use despite substantial substance-related problems. Core features include impaired control over substance use, tolerance, withdrawal, and compulsive drug-taking behavior [1]. SUD is also associated with cognitive impairment, particularly in executive functioning, attention, and decision-making, which may contribute both to the development of the disorder and to relapse risk [2–4]. As the prevalence of SUD has increased in recent years, it remains a major public health concern [5–6].

Psychiatric comorbidity is common in SUD, and attention-deficit/hyperactivity disorder (ADHD) is among the most prevalent co-occurring conditions. Approximately 21%-23% of patients with SUD meet DSM criteria for comorbid ADHD [7–8]. ADHD is a neurodevelopmental disorder characterized by persistent inattention and/or hyperactivity/impulsivity that interferes with daily functioning [1]. In adulthood, overt hyperactivity often declines, whereas inattention and executive dysfunction tend to become more prominent [9–10]. Adults with ADHD are at substantially increased risk of developing SUD [11], and adolescents with ADHD tend to initiate substance use earlier than their peers without ADHD [12–13]. Screening for adult ADHD in addiction care has been widely recommended [14], and early detection may improve treatment planning and outcomes. At the same time, ADHD appears to be both overrepresented and underdiagnosed in addiction treatment settings, which may further complicate clinical management [14–15].

Co-occurring SUD and ADHD are associated with a more severe clinical presentation than either disorder alone. Individuals with both conditions are more likely to develop substance-related problems at a younger age, to use a broader range of substances [16–18], and to experience poorer treatment outcomes and a greater overall psychiatric burden [7, 19].

Most individuals with SUD do not use a single substance exclusively. Instead, many use multiple substances either simultaneously or sequentially over time [5, 20]. Polysubstance use represents a particularly severe and clinically important pattern of substance use and is associated with greater addiction severity, poorer treatment retention, increased relapse risk, and higher mortality than single-substance use [5, 21–24].

In Europe, methylphenidate (MPH) is considered a first-line pharmacological treatment for moderate-to-severe adult ADHD [25–26]. MPH is a central nervous system stimulant that blocks the reuptake of dopamine and norepinephrine, thereby increasing synaptic concentrations of these neurotransmitters. In adults with ADHD without SUD, stimulant treatment has been shown to reduce core ADHD symptoms effectively [27–29]. However, the evidence is less robust in individuals with co-occurring ADHD and SUD. Previous studies suggest that MPH improves ADHD symptoms to some extent in this population but has limited or no effect on substance use outcomes [30–31]. Meta-analyses have generally found small but positive effects on ADHD symptoms in individuals with comorbid ADHD and current SUD [32].

An important consideration in this context is the potential influence of placebo and expectancy effects [33]. A recent systematic review and meta-analysis reported a substantial placebo response in ADHD, corresponding to an approximately 23% reduction in self-reported symptoms [34]. Self-report remains the most common method for assessing ADHD symptoms in adults, although semi-structured interviews, collateral informant reports, and neuropsychological measures are also used. Across psychiatric and neurocognitive research, expectations have been shown to influence symptom ratings and perceived treatment effects [35–36]. This may be particularly relevant in populations with complex comorbidity and extensive prior experience with psychoactive substances.

Previous studies have largely focused on individuals with single-substance use, leaving limited knowledge about those with polysubstance use. This gap is clinically important, as polysubstance use is common and often associated with greater severity. The present study therefore aimed to examine the acute effects of immediate-release methylphenidate (IR-MPH) versus placebo on cognitive performance in adults with untreated ADHD and longstanding polysubstance use, while also exploring self-rated expectations and perceived performance. We hypothesized that both IR-MPH and placebo would improve cognitive performance, consistent with possible expectancy-related effects in this population.

## Method

### Participants

Participants were adult outpatients with SUD and ADHD recruited from the Dependency Unit at the Psychiatric Clinic in the Country of Västmanland, Sweden between July 2016 and June 2021. At the time of recruitment, all participants had a history of polysubstance use for at least 10 years. Concurrent use of multiple substances was reported by all participants during this period. Fourteen participants had regularly used 4–6 substances simultaneously, 16 participants reported 3 substances simultaneously, and 9 participants reported 2 substances simultaneously during the years. The substances most commonly used were amphetamine (26 participants), cannabinoids (24 participants). benzodiazepine derivatives (23 participants), opioids (22 participants), cocaine (11 participants), and ectasy (5 participants). In addition to these, various types of synthetic cathinones were also reported. Four participants had ongoing opioid agonist therapy. Shortly before study participation, all participants had been diagnosed with comorbid ADHD by clinicians specialized in neurodevelopmental disorders.

ADHD diagnoses were established according to DSM-5 criteria and included assessment with the Diagnostic Interview for ADHD in Adults – DIVA 2.0 [37], the Mini International Neuropsychiatric Interview – M.I.N.I [38]., the Adult ADHD Self-Report Scale – ASRS [39], and the Behavior Rating Inventory of Executive Function-Adult Version – BRIEF-A [40]. Childhood ADHD symptoms were assessed using the Wender Utah Rating Scale – WURS [41]. Collateral information from significant others was obtained through telephone interviews.

None of the participants had previously received treatment for a neurodevelopmental disorder. However, 16 participants had previously been treated for mild anxiety, depression, or post-traumatic stress disorder. Among these, 8 participants were currently receiving antidepressant medication. Inclusion criteria were age 18 years or older, fulfillment of DSM-5 criteria for at least two non-tobacco substance use disorders in addition to ADHD, and a Qb-score of at least 1.3 on one or more of the three cardinal QbTest parameters. The main exclusion criteria were alcohol use, use of non-prescribed medication, or illicit drug use during the preceding month; current severe untreated somatic or psychiatric illness; resting heart rate > 100 beats/min; blood pressure > 150/95 mmHg; and positive pregnancy test.

### Randomization and masking

The randomization sequence was generated by a research nurse at the Centre for Clinical Research, County of Västmanland, Sweden. A pharmacist at the local pharmacy prepared, blinded, and labeled the study medication, ensuring that active medication and placebo were identical in appearance. Neither the research nurse nor the pharmacist was involved in any other aspect of the study.

### Objective outcome measures

Objective outcomes were assessed using the QbTest, a computerized continuous performance test designed to measure the three core symptom domains of ADHD: activity, inattention, and impulsivity. These domains are presented in the test report as the cardinal parameters QbActivity, QbInattention, and QbImpulsivity [42–43]. Qb-scores are normalized standard scores (z-scores) adjusted for age and gender, with higher scores indicating greater symptom severity. Performance is classified as within normal limits when the Qb-score falls between − 1.0 and 1.0; scores ranging from 1.1 to 1.4 are regarded as mildly atypical, whereas scores of ≥ 1.5 are interpreted as markedly divergent. A change in Qb-score of at least 0.5 points is considered clinically meaningful, with decreases indication improvement and increases indicating worsening [43]. Published psychometric data indicate a sensitivity of 86% and a specificity of 83% [44]. The standard administration time for the QbTest is 20 min.

QbActivity is derived from the four parameters: Time Active, Distance, Area and Microevents. Time Active represents the proportion of the test duration during which the participant´s movement exceeded 1 cm/s (0.4 inches/s). Distance denotes the total path length traversed by a reflective marker, expressed in meters. Area refers to the spatial extent covered by the headband-mounted reflector throughout the assessment. A Microevent is recorded whenever the positional displacement of the marker exceeds 1 mm relative to the preceding Microevent.

QbInattention comprises four parameters: Omission Errors, Reaction Time (RT), Reaction Time Variation, and Normalized Variation. An omission error is registered when the participant fails to respond to a target stimulus (i.e. omits a required button press). RT is calculated as the mean latency between stimulus onset and correct button press, measured in milliseconds. Reaction Time Variation corresponds to the standard deviation of RT across valid trials.

QbImpulsivity is operationalized through Commission Errors. A commission error occurs when a response is erroneously executed following a non-target stimulus (i.e. an inappropriate button press). The overall Error Rate quantifies the frequency of incorrect responses, encompassing both erroneous presses to non-targets and failures to respond to targets.

### Subjective outcome measures

Visual analog scales (VASs) were used to assess participants’ self-rated expected performance (“How do you think you will perform on the QbTest?”), mental effort (“How exhausting was it to complete the QbTest?”), perceived performance (“How do you think you performed on the QbTest?”), and perceived help from the study medication (“How much help do you think you received from the medication?”). Participants also rated perceived difficulty in understanding the task (“How difficult was it to understand the task?”). Each VAS consisted of a 100-mm line with verbal anchors at each endpoint (e.g. “Not well at all” to “Very well,” “Very exhausting” to “Not exhausting at all,” “No help at all” to “Very much help,” and “Very difficult” to “Not difficult at all”).

### Procedure

**Fig. 1:**
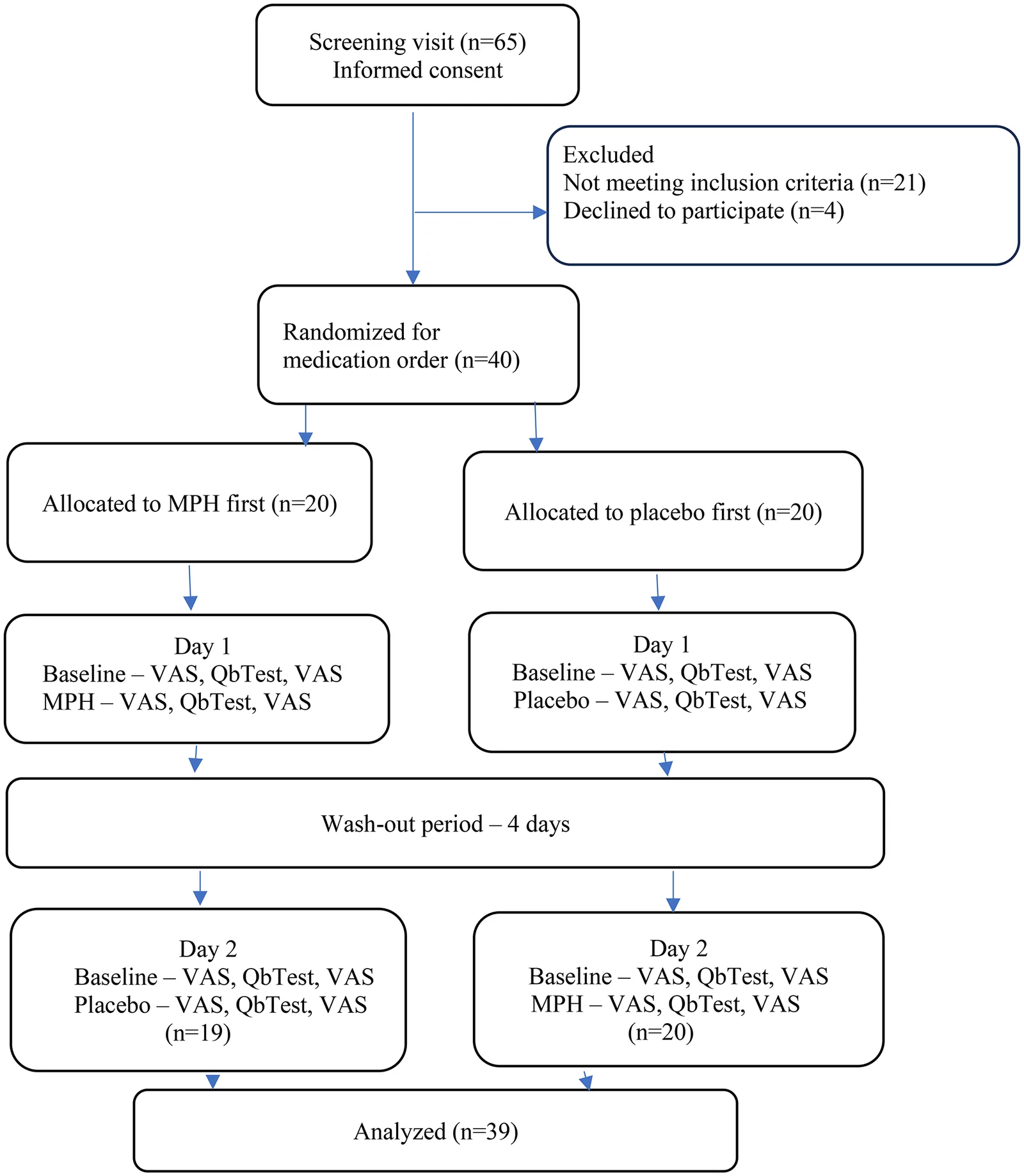
Flowchart of the procedure and randomization of the study. MPH = Methylphenidate Immideate release, VAS = Visual analogue scale, n=group size

After the screening visit, during which eligibility criteria were assessed and the QbTest was administered, participants were consecutively enrolled and randomized to one of two treatment sequences: MPH-first or placebo-first. At each study visit at the Psychiatric Clinic, participants were screened for alcohol and drug use; female participants were also screened for pregnancy. The study used a randomized, double-blind, placebo-controlled crossover design (Fig. 1).

**Table 1:** Clinical and demographic characteristics for participants when included in the study (*n* = 39)

*Characteristic*	MPH-first Group	Placebo-first Group	Total
	(*n*=19)	(*n*=20)	(*n*=39)
*Gender*			
Male/Female (n)	13/6	15/5	28/11
Age [mean years (SD)]	37.2 (10.4)	34.2 (9.9)	35.8 (10.1)
Height cm [mean (SD)]	177.89 (9.41)	175.06 (8.91)	176.44 (9.15)
Weight kg [mean (SD)]	94.72 (15.77)^*^	81.77 (16.29)	88.08 (17.13)
*Work*			
Employee (n)	6	7	13
Unemployed (n)	13	13	26
Smoking cigarettes (n)	10	16	26
Snuff (n)	14	13	27
QoL^1^ [mean (SD)]	47.79 (24.37)	51.75 (26.53)	49.82 (24.24)
QbHyperactivity^2^ [Z-mean (SD)]	2.29 (1.15)	2.30 (1.04)	2.30 (1.08)
QbInattention^2^ [Z-mean (SD)]	1.62 (1.09)	1.39 (1.45)	1.50 (1.28)
QbImpulsivity^2^ [Z-mean (SD)]	1.30 (1.49)	1.02 (1.73)	1.16 (1.60)
*ASRS*^*3*^ [mean [SD])			
ASRS Hyperactivity/Impulsivity	24.05 (5.52)	20.00 (7.33)	21.97 (6.74)
ASRS Inattention	24.47 (3.69)	21.85 (6.58)	23.13 (5.46)
ASRS Total	48.53 (7.77)	41.85 (13.35)	45.10 (11.36)
*BRIEF-A*^*4*^ [T-mean (SD)]			
Inhibit	75.16 (9.24)	76.65 (9.47)	75.92 (9.27)
Shift	75.05 (12.08)	70.80 (11.32)	72.87 (11.74)
Emotional Control	68.63 (11.40)	64.95 (11.47)	66.74 (11.44)
Self-Monitor	70.21 (13.97)	72.80 (11.62)	71.54 (12.71)
Behavioral Regulation Index (BRI)	76.63 (11.54)	74.95 (11.74)	75.77 (11.52)
Initiate	73.53 (8.29)	68.05 (9.12)	70.72 (9.04)
Working Memory	77.42 (8.32)	77.55 (6.95)	77.49 (7.55)
Plan/Organize	73.00 (8.04)	73.10 (9.56)	73.05 (8.74)
Task Monitor	71.58 (8.21)	70.55 (10.57)	71.05 (9.39)
Organization of Materials	65.16 (10.16)	60.50 (9.01)	62.77 (9.74)
Metacognition Index (MI)	75.53 (5.85)	73.00 (7.32)	74.23 (6.68)
Global Executive Composit (GEC)	78.10 (7.58)	75.75 (9.12)	76.90 (8.38)

SD = Standard deviation. ^1^ QoL = Self-reported Quality of life, assessed by a Visual Analogue Scale. ^2^ QbTest at screening visit = The normalized standard scores for the cardinal symptoms hyperactivity, inattention and impulsivity. ^3^ASRS = the Adult Self-Report Scale. ^4^ BRIEF-A = the Behavioral Rating Inventory of Executive Functions – Adults. ^*^p < 0.05

**Table 2:** Comparisons of Qb-scores between baseline, IR-MPH and placebo (*n* = 39)

	Baseline vs. IR-MPH^1^			Baseline vs. placebo			IR-MPH^1^ vs. placebo		
	Mean difference (95% CI)	p	d^2^	Mean difference (95% CI)	p	d^2^	Mean difference (95% CI)	p	d^2^
*Cardinal parameters*									
QbActivity^3^	0.83 (0.37–1.28)	0.001	0.65	0.15 (-0.12-0.41)	0.829	0.11	0.68 (0.27–1.08)	0.002	0.81
QbInattention^3^	1.00 (0.55–1.45)	0.001	0.81	0.35 (-0.02-0.72)	0.072	0.25	0.65 (0.30–1.01)	0.001	0.70
QbImpulsivity^3^	0.83 (0.12–1.55)	0.015	0.60	0.00 (-0.59-0.60)	1.00	0.01	0.86 (0.30–1.42)	0.004	0.58
*Activity measures*									
Time Active (%)	13.87 (3.87–23.87)	0.003	0.62	5.92 (-0.50-12.34)	0.086	0.27	7.95 (0.81–15.07)	0.030	0.42
Distance (meter)	6.58 (2.54–10.61)	0.001	0.84	3.16 (-0.02-6.34)	0.052	0.33	3.41 (1.25–5.58)	0.003	0.42
Area (cm)	27.95 (11.72–44.17)	0.001	0.84	9.97 (-2.86-22.81)	0.221	0.24	17.69 (8.03–27.36)	0.001	0.71
*Attention & Impulsive measures*									
Reaction Time (ms)	50.69 (27.24–74.14)	0.001	0.43	35.15 (2.37–67.84)	0.029	0.27	15.54 (-14.64-45.72)	0.304	0.21
Reaction Time Variation (ms)	43.00 (20.88–65.12)	0.001	0.77	18.20 (-3.37-39.78)	0.145	0.31	24.79 (5.18–44.41)	0.015	0.51
Omission Error (%)	18.30 (10.66-25-95)	0.001	1.06	3.81 (-1.82-9.44)	0.405	0.20	14.49 (7.21–21.78)	0.001	0.96
Commission Error (%)	1.82 (-0.29-3.92)	0.127	0.54	0.46 (-0.96-1.89)	1.00	0.18	1.35 (0.46–2.24)	0.004	0.34
Error Rate (%)	5.89 (3.38–8.40)	0.001	1.17	1.34 (-0.51-3.19)	0.303	0.26	4.55 (2.76–6.34)	0.001	0.92

^1^ Immediate-release methylphenidate, ^2^ Effect size (Cohen´s d), ^3^ Qb = Standard scores

**Table 3:** Means and 95% CI for self-reported assessments before and after IR-MPH/placebo (*n* = 39)

Variable	Pre-IR-MPH^1^	Post IR-MPH^2^	Pre-placebo^3^	Post-placebo^4^
*Self-reported outcomes* ^*5*^				
Expected performance	53.61 [46.77–60.46]	65.49 [59.35–71.63]^.003^	54.46 [47.89–61.04]	58.31 [51.37–65.25]
Perceived performance	50.13 [42.79–57.47]	77.56 [71.35 83.78]^.001^	50.54 [41.74–59.33]	59.18 [50.74–67.62]
Mental effort^6^	79.03 [74.97–83.08]	42.51 [33.44–51.59]^.001^	77.82 [72.19–83.45]	58.59 [49.51–67.67]^.001^
Task severity^7^	25.92 [17.43–34.42]	16.18 [10.19–22.16]	26.31 [17.84–34.77]	21.64 [13.94–29.34]
Perc benef medication^8^		68.18 [59.33–77.03]^.001^		37.54 [27.76–47.31]

^1^ Baseline – before IR-MPH, ^2^ After immediate-release methylphenidate, ^3^ Baseline-before placebo, ^4^ After placebo, ^5^ Visual analogue scales (1–100 mm),

^6^ Perceived mental effort, ^7^ perceived difficulty understanding the task, ^8^ Perceived benefit from the study medication

**Table 4:** Means and standard deviations for discrepancy scores across baseline, IR-MPH, and placebo conditions for self-rated expected performance relative to reverse-coded omission errors, perceived performance relative to reverse-coded omission errors, and expected performance relative to perceived performance (*n* = 39)

Variable	Baseline	IR-MPH^1^	Placebo
Expect perform relative to omission errors^2^	27.5 (30.7)	-25.3 (22.2)	-20.8 (20.2)
Perceived performance relative to omission errors^2^	24.9 (34.8)	-13.3 (22.9)	-19.9 (22.4)
Expected performance relative to perceived performance	2.7 (20.8)	-12.1 (23.0)	-0.9 (20.8)

^1^ Immediate-release methylphenidate, ^2^ Negative values indicate lower self-rated expected or perceived performance relative to reverse-coded omission errors (100 = 0% omission error), and lower expected performance relative to perceived performance

The study comprised two experimental days separated by a 4-day washout period. On each study day, participants completed the QbTest before and 80 min after intake of either a single 20-mg dose of IR-MPH (Medikinet^®^) or a placebo containing an inert substance, according to the randomized sequence. Before each QbTest, participants viewed an instructional video, received standardized oral instructions, and rated their expected performance on a VAS. After each QbTest, they rated mental effort, perceived performance, and task comprehension. Following the second post-dose test, they also rated the perceived benefit of the medication. All QbTests were administered in the morning to minimize time-of-day effects.

### Statistical analyses

Repeated-measures analyses of variance (ANOVAs), followed by Bonferroni-adjusted post hoc comparisons, were used to examine differences across four conditions: pre-IR-MPH, post-IR-MPH, pre-placebo, and post-placebo. The efficacy of IR-MPH relative to placebo was assessed by comparing change scores between conditions using paired-samples t-tests. Effect sizes were calculated as Cohen’s d_*z*_ for paired samples, with values of 0.20, 0.50, and 0.80 interpreted as small, medium, and large effects, respectively. Paired-samples t-tests were also used to examine potential sequence and period effects on the QbTest performance.

Discrepancies between subjective self-ratings (expected and perceived performance) and objective QbTest performance were also examined. Objective performance was indexed using the reverse-coded omission error parameter (100 = 0% omission error) from the QbTest. Test-retest reliability between the two baseline (pre-tablet) conditions was estimated using intraclass correlation coefficients (ICCs) from a two-way mixed-effects model with absolute agreement. All analyses were performed using IBM SPSS Statistics for Windows, version 31.0.

A sample size of 36 participants was estimated to detect a clinically meaningful improvement in QbTest performance, defined as a reduction in Qb-score of at least 0.5, with 80% power at a two-sided alpha level of 0.05. In total, 40 participants were enrolled in the study. However, one participant discontinued after the first study day due to COVID-19.

## Results

Thirty-nine adults participated in the study, including 28 males (mean age 36.4) and 11 females (mean age 33.6). The participants’ educational background were as follows; incomplete primary education (*n* = 6), completed primary education (*n* = 7), incomplete secondary education (*n* = 5), completed secondary education (*n* = 9), and completed vocational education (*n* = 12). All participants met DSM-5 criteria for both SUD and ADHD. Of the total sample, 22 participants (56.4%) met criteria for the combined ADHD presentation, 15 (38.5%) for the hyperactive/impulsive presentation, and 2 (5.1%) for the inattentive presentation. All participants received a single 20-mg dose of IR-MPH, corresponding to a mean dose of 0.24 mg/kg (range 0.16–0.36). Clinical and demographic characteristics at study entry are presented in Table 1. No significant gender differences were observed.

### Objective outcomes

#### Efficacy measures

Compared with baseline, participants showed significant improvement after IR-MPH on all three cardinal QbTest parameters. QbActivity decreased from a baseline mean of 2.27 (SD = 1.27) to 1.45 (SD = 1.25) after IR-MPH (*p* < 0.001). QbInattention decreased from a baseline mean of 1.17 (SD = 1.22) to 0.16 (SD = 1.28) after IR-MPH (*p* < 0.001). QbImpulsivity was reduced from a mean of 0.93 (SD = 1.58) to 0.10 (SD = 1.15) after IR-MPH (*p* < 0.015). These improvements remained significant after adjustment for placebo effects. No statistically significant results were found after placebo compared with baseline on any of the three cardinal QbTest parameters. QbActivity showed a baseline mean of 2.09 (SD = 1.35) and a mean of 1.95 (SD = 1.19) after placebo (*p* = 0.829). QbInattention showed a baseline mean of 1.21 (SD = 1.37) and a mean of 0.86 (SD = 1.42) after placebo (*p* = 0.072). QbImpulsivity showed a baseline mean of 0.74 (SD = 1.55) and a mean of 0.75 (SD = 1.35) after placebo (*p =* 1.0).

#### IR-MPH versus baseline for the primary outcome measures

After IR-MPH, significant improvements were observed across all primary outcomes measures with the exception of commission errors. Time active decreased from a baseline mean of 34.95% (SD = 23.57) to a mean of 21.08% (SD = 21.38) after IR-MPH (*p* < 0.003). Distance decreased from a mean of 14.59 m (SD = 10.05) at baseline to 8.01 m (SD = 4.56) after IR-MPH (*p* < 0.001), and Area decreased from a mean of 56.10 cm^2^ (SD = 42.16) to 28.15 cm^2^ (SD = 20.45) after IR-MPH (*p* < 0.001).

Among cognitive performance measures, omission errors decreased substantially from a baseline mean of 27.47% (SD = 20.98) to 9.17% (SD = 12.60) after IR-MPH (*p* < 0.001). Mean reaction time shortened from 604.48 ms (SD = 125.74) to 553.79 ms (SD = 111.42; *p* < 0.001), and reaction time variation decreased from 171.15 ms (SD = 55.02) to 128.15 ms (SD = 56.39) after IR-MPH (*p* < 0.001). Error rate declined from 8.76% (SD = 6.25) to 2.86% (SD = 3.43) after IR-MPH (*p* < 0.001). Commission errors showed a numerical reduction from a baseline mean of 2.58% (SD = 4.69) to 0.76% (SD = 0.90) after IR-MPH; however, this change did not reach statistical significance (*p* = 0.127).

#### Placebo versus baseline for the primary outcome measures

After placebo, a significant improvement was observed only for reaction time. Mean reaction time decreased from 613.05 ms (SD = 131.53) at baseline to 577.89 ms (SD = 125.57) after placebo (*p* = 0.029). No significant changes were observed for the remaining outcome measures; time active (baseline mean = 31.95%, SD = 24.09; after placebo mean = 26.03%, SD = 19.22; *p* = 0.086), distance (baseline mean = 14.42 m, SD = 11.17; after placebo mean = 11.26 m, SD = 7.25; *p* = 0.052), area (baseline mean = 53.97 cm^2^, SD = 46.80; after placebo mean = 44.00 cm^2^, SD = 32.82; *p* = 0.221), omission errors (baseline mean = 24.64%, SD = 18.36; after placebo mean = 20.83%, SD = 19.63; *p* = 0.405), reaction time variation (baseline mean = 175.79 ms, SD = 57.57; after placebo mean = 157.59 ms, SD = 59.68; *p* = 0.145), commission errors (baseline mean = 1.89%, SD = 3.23; after placebo mean = 1.43%, SD = 1.73; *p* > 0.99), and error rate (baseline mean = 7.68%, SD = 5.17; after placebo mean = 6.34%, SD = 5.05; *p* = 0.303). No significant sequence or gender effects were observed for the objective outcomes.

#### Comparison of IR-MPH and placebo effects

The magnitude of improvement was consistently greater following IR-MPH compared with placebo across all assessed measures (Table 2). For time active, the reduction was 13.87% with IR-MPH versus 5.92% with placebo. Similarly, distance decreased by 6.58 m after IR-MPH compared with 3.16 m after placebo, and area decreased by 27.95 cm^2^ versus 9.97 cm^2^, respectively. For omission errors, the reduction was 18.30% after IR-MPH compared with 3.81% after placebo. Although both conditions produced statistically significant reductions in mean reaction time, the magnitude of improvement was greater with IR-MPH (50.69 ms) than with placebo (35.15 ms). Reaction time variation decreased by 43.00 ms after IR-MPH but by only 18.20 ms after placebo. Error rate showed a marked reduction of 5.89% points following IR-MPH compared with 1.34% points following placebo. Neither condition produced a statistically significant change in commission errors. These findings indicate that IR-MPH exerted broad beneficial effects on motor activity, sustained attention, and response variability, whereas placebo effects were limited to a modest improvement in reaction time.

### Subjective outcomes

#### Expected performance

Expected performance increased significantly following IR-MPH compared with baseline [mean difference 11.87, 95% CI (3.21–20.53), *F* (3, 114) = 6.05, *p* = 0.003], whereas no significant change was observed following placebo (Table 3). No significant sequence or gender effects were found.

#### Perceived performance

Participants reported significantly improved perceived performance following IR-MPH compared with baseline [mean difference 27.44, 95% CI (14.05–40.82), *F* (2.53, 96.09) = 15.51, *p* < 0.001]. No significant change was observed following placebo. However, participants randomized to the placebo-first sequence reported higher perceived performance after placebo than those randomized to the IR-MPH-first sequence [mean difference 18.49, 95% CI (0.69–36.29), *t* (37) = 3.10, *p* = 0.042]. No significant gender differences were observed.

#### Mental effort

Participants reported significantly lower mental effort following both IR-MPH and placebo, *F* (2.40, 91.36) = 26.83, *p* < 0.001. Compared with baseline, mental effort was lower after IR-MPH [mean difference 36.51, 95% CI (22.34–50.69), *p* < 0.001] and after placebo [mean difference 19.23, 95% CI (6.78–31.68), *p* < 0.001]. No significant sequence or gender effects were observed.

#### Perceived benefit from the study medication

Perceived benefit differed significantly between the study medications, with participants reporting greater perceived benefit from IR-MPH than from placebo [mean difference 30.64, 95% CI (17.93–43.30), *t* (37) = 4.88, *p* < 0.001]. No significant sequence or gender effects were observed.

#### Difficulty understanding the task

Participants reported little difficulty in understanding the task [mean 26.30, 95% CI (17.84–34.77)]. No significant sequence or gender differences were observed.

#### Discrepancies between self-ratings and objective outcomes

Table 4 presents means and standard deviations for discrepancy scores at baseline and following IR-MPH and placebo for expected performance relative to reverse-coded omission errors, perceived performance relative to reverse-coded omission errors, and expected performance relative to perceived performance.

There was a significant main effect of condition on the discrepancy between expected performance and omission errors, *F* (1.49, 56.72) = 85.12, *p* < 0.001. Compared with baseline, mean differences were 52.90, 95% CI (40.37–65.43), *p* < 0.001 after IR-MPH, and 48.41, 95% CI (35.37–61.45), *p* < 0.001 after placebo.

Condition also had a significant effect on the discrepancy between perceived performance and omission errors, *F* (1.51, 57.42) = 31.31, *p* < 0.001. Compared with baseline, mean differences were 38.16, 95% CI (20.25–56.06), *p* < 0.001 after IR-MPH, and 44.87, 95% CI (28.13–61.61), *p* < 0.001 after placebo.

A significant main effect of condition was also observed for the discrepancy between expected and perceived performance, *F* (2, 76) = 4.41, *p* = 0.015. Compared to baseline,

mean difference 14.74, 95% CI (1.03–28.45), *p* = 0.031 after IR-MPH.

#### Test-retest reliability

Intraclass correlation coefficients (ICCs) were calculated to assess reliability between the two baseline measurements. For the subjective measures, ICCs were 0.59 for expected performance, 0.48 for mental effort, 0.84 for perceived performance, and 0.90 for difficulty understanding the task. For objective measures, ICCs were 0.93 for QbActivity, 0.92 for QbInattention, 0.82 for QbImpulsivity, and 0.93 for omission errors. No QbTest order effects were observed between the two study days. All ICCs were statistically significant (*p* < 0.05).

## Discussion

The present study examined the acute effects of IR-MPH versus placebo on objectively measured QbTest performance in adults with untreated ADHD and longstanding polysubstance use. Overall, IR-MPH was associated with significant improvement across all three cardinal QbTest parameters – QbActivity, QbInattention, and QbImpulsivity – whereas placebo was associated with improvement only in reaction time. Direct comparisons further showed that IR-MPH was superior to placebo on all objective QbTest outcomes except reaction time. Together, these findings suggest that acute IR-MPH may improve cognitive performance on the QbTest in this clinically complex population, whereas placebo effects on objective performance appear to be more limited.

These findings are broadly consistent with previous studies showing that a single dose of IR-MPH improves QbTest performance in children [45], adolescents [46], and adults with ADHD without SUD [47–48]. The present results extend this literature by indicating that similar acute effects may also be observed in adults with ADHD and longstanding polysubstance use. At the same time, the placebo response appeared more limited than that reported in some previous studies of adults with ADHD without SUD, in which placebo was associated with broader improvement across objective performance measures [47]. This difference may suggest that practice or expectancy effects operate differently in individuals with co-occurring SUD, although this interpretation should be considered tentative.

An additional finding was that placebo was associated with improvement in reaction time but not with broader improvement in attentional regulation, as no corresponding effects were observed for reaction time variation, omission errors, or total error rate. One possible interpretation is that placebo primarily affected non-specific alertness, arousal, or test familiarity rather than core ADHD-related cognitive control. Similarly, the absence of a significant effect of IR-MPH on commission errors may indicate that certain aspects of inhibitory control were less sensitive to acute treatment in this sample, or that variation in this measure reduced statistical power.

The subjective findings add an important perspective to the objective results. Perceived mental effort decreased after both IR-MPH and placebo, whereas expected performance, perceived performance, and perceived benefit improved more clearly after IR-MPH. Similar findings have also been reported in a study of adults with ADHD without comorbid SUD [49]. This pattern suggests that subjective experience during cognitive testing may reflect both pharmacological and expectancy-related influences. In particular, the reduction in mental effort after placebo, despite limited change in objective performance, indicates that self-reported benefit should be interpreted cautiously in populations in which expectations regarding stimulant medication may be strong.

The discrepancy analyses further highlight the distinction between subjective and objective outcomes. At baseline, participants overestimated their performance, as both expected and perceived performance ratings were more favourable than their omission error results. After both IR-MPH and placebo, this pattern reversed, with participants rating their expected and perceived performance less favourable than their actual omission error performance indicated. Notably, the discrepancy between expected and perceived performance changed significantly only after IR-MPH, with perceived performance exceeding prior expectations. This may indicate that IR-MPH produced a subjectively salient benefit that was not fully captured by pre-test expectations alone. More broadly, these findings suggest that self-evaluations of cognitive performance are dynamic and do not necessarily map closely onto objective test results.

These observations may have clinical relevance, as the assessment and follow-up of adult ADHD often rely heavily on self-report. In patients with co-occurring SUD, subjective ratings may be influenced by expectations, prior expectations with psychoactive substances, and situational factors relating to testing. The present findings therefore support the inclusion of objective measures as a complement to self-report when evaluating early medication response in clinically complex ADHD populations.

### Limitations and strengths

Several limitations should be considered. First, the study assessed acute effects only, and the findings cannot be generalized to longer-term treatment response, optimal dosing, tolerability, or substance use outcomes. Second, although the QbTest provides standardized objective data, no single test can fully capture the complexity of everyday ADHD-related impairment. Third, due to administrative reasons, no cognitive assessment data were available for the participants. The study also has important strengths. The randomized, double-blind, placebo-controlled crossover design allowed participants to serve as their own controls and strengthened the comparison between active medication and placebo. The inclusion of both objective and subjective outcome measures made it possible to examine not only performance change but also participants’ expectations and perceived benefit. In addition, the use of two baseline assessments, one for each trial day, served multiple purposes; (1) to establish reference values for calculating condition-specific change scores; (2) to evaluate and adjust for potential learning or practice effects associated with cognitive testing, as earlier studies have reported better performance during the second administered CPT [50–51], although randomization of stimulus sequences within the QbTest further reduce such effects [43]; and (3) to examine potential sequences or carryover effects, although these are theoretically unlikely with IR-MPH given its brief pharmacological half-life. Test-retest reliability between the two daily baselines was high, consistent with previous reports [52]. Given evidence that pharmacological response may be most pronounced among individuals with greater baseline impairment [53–54], we applied a more inclusive threshold (Qb-score ≥ 1.3) than the clinical cutoff (Qb-score ≥ 1.5) indicating a atypical score [43]. This approach was intended to minimize regression to the mean artifacts that can arise when extreme baseline scores naturally shift toward the population mean upon retesting. However, this did not result in any participant screened with QbTest had to be excluded due to low Qb-score. A further strength is the use of data from a clinical setting and focus on a understudied population with both untreated ADHD and longstanding polysubstance use. None of the participants had previously participated in any clinical study.

## Conclusion

In conclusion, IR-MPH was associated with short-term improvements in objective QbTest performance in adults with untreated ADHD and longstanding polysubstance use, whereas placebo effects were largely limited to reaction time and subjective experience. These findings support the inclusion of objective assessments alongside self-report when evaluating early treatment response in adults with ADHD and co-occurring SUD. Further studies are needed to determine whether these acute effects translate into sustained clinical benefit during longer-term treatment.

## Data Availability

The datasets generated and/or analyzed during the current study are not publicly available due to protection of participants’ confidentiality but are available from the corresponding author on reasonable requests.

## Abbreviations

ADHD: Attention-deficit/hyperactivity disorder
ANOVA: Analysis of variance
ASRS: The Adult ADHD Self-Report Scale
BRIEF-A: The Behavior Rating Inventory of Executive Function-Adult Version
CI: Confidence Interval
DIVA: The Diagnostic Interview for ADHD in Adults
DSM-5: Diagnostic and Statistical Manual of Mental Disorders (5th edition)
ICC: Intraclass Correlation Coefficient
IR-MPH: Immediate-release methylphenidate
MINI: The Mini International Neuropsychiatric Interview
MPH: Methylphenidate
SD: Standard Deviation
SUD: Substance use disorder
VAS: Visual Analogue Scale
WURS: The Wender Utah Rating Scale
